# Online Doctor Reviews: Do They Track Surgeon Volume, a Proxy for Quality of Care?

**DOI:** 10.2196/jmir.2005

**Published:** 2012-04-10

**Authors:** Jeffrey Segal, Michael Sacopulos, Virgil Sheets, Irish Thurston, Kendra Brooks, Ryan Puccia

**Affiliations:** ^1^Medical Justice Services, Inc.Greensboro, NCUnited States; ^2^Sacopulos, Johnson, SacopulosTerre Haute, INUnited States; ^3^Indiana State UniversityDepartment of PsychologyTerre Haute, INUnited States; ^4^Nussbaum Entrepreneurial CenterGreensboro, NCUnited States; ^5^Case Western Reserve UniversityCleveland, OHUnited States

**Keywords:** Doctor review, rating websites, physician reviews, online reputation, clinical outcomes, selection of physicians, surgical volume, surgeon volume

## Abstract

**Background:**

Increasingly, consumers are accessing the Internet seeking health information. Consumers are also using online doctor review websites to help select their physician. Such websites tally numerical ratings and comments from past patients. To our knowledge, no study has previously analyzed whether doctors with positive online reputations on doctor review websites actually deliver higher quality of care typically associated with better clinical outcomes and better safety records.

**Objective:**

For a number of procedures, surgeons who perform more procedures have better clinical outcomes and safety records than those who perform fewer procedures. Our objective was to determine if surgeon volume, as a proxy for clinical outcomes and patient safety, correlates with online reputation.

**Methods:**

We investigated the numerical ratings and comments on 9 online review websites for high- and low-volume surgeons for three procedures: lumbar surgery, total knee replacement, and bariatric surgery. High-volume surgeons were randomly selected from the group within the highest quartile of claims submitted for reimbursement using the procedures’ relevant current procedural terminology (CPT) codes. Low-volume surgeons were randomly selected from the lowest quartile of submitted claims for the procedures’ relevant CPT codes. Claims were collated within the Normative Health Information Database, covering multiple payers for more than 25 million insured patients.

**Results:**

Numerical ratings were found for the majority of physicians in our sample (547/600, 91.2%) and comments were found for 385/600 (64.2%) of the physicians. We found that high-volume (HV) surgeons could be differentiated from low-volume (LV) surgeons independently by analyzing: (1) the total number of numerical ratings per website (HV: mean = 5.85; LV: mean = 4.87, *P*<.001); (2) the total number of text comments per website (HV: mean = 2.74; LV: mean = 2.30, *P*=.05); (3) the proportion of glowing praise/total comments about quality of care (HV: mean = 0.64; LV: mean = 0.51, *P*=.002); and (4) the proportion of scathing criticism/total comments about quality of care (HV: mean = 0.14; LV: mean = 0.23, *P*= .005). Even when these features were combined, the effect size, although significant, was still weak. The results revealed that one could accurately identify a physician’s patient volume via discriminant and classification analysis 61.6% of the time. We also found that high-volume surgeons could not be differentiated from low-volume surgeons by analyzing (1) standardized z score numerical ratings (HV: mean = 0.07; LV: mean = 0, *P*=.27); (2) proportion of glowing praise/total comments about customer service (HV: mean = 0.24; LV: mean = 0.22, *P*=.52); and (3) proportion of scathing criticism/total comments about customer service (HV: mean = 0.19; LV: mean = 0.21, *P*=.48).

**Conclusions:**

Online review websites provide a rich source of data that may be able to track quality of care, although the effect size is weak and not consistent for all review website metrics.

## Introduction

Every day a patient somewhere will ask: “Is Dr. X a good doctor?”

By itself, such a statement is meaningless. The patient is really asking if Dr. X is a good doctor for a particular end. For example, is Dr. X a good doctor to address a particular symptom or to perform a defined treatment?

As an analogy, the question is as unspecific as “Is this a good car?” Better questions are: “Is this a good car for the gas mileage?” or “Is this a good car for value?” or “Is this a good car for accelerating quickly?” Each question delivers a different answer.

Patients access the Internet seeking an answer to the question “Is Dr. X a good doctor?” but they are really asking if Dr. X is a good doctor for a particular end. Is Dr. X a good diagnostician? Or is he compassionate with excellent listening skills? Or is she a doctor who has treated over 1000 patients with Chiari malformation? A typical doctor review website rarely makes that type of distinction with sufficient clarity.

Our hypothesis is that isolated doctor review websites may not be good proxies for what patients truly care about—namely clinical outcomes and safety. Doctor review websites measure whether patients like their doctor. These websites also measure subjective responses. Does the doctor communicate well? Does the doctor listen? How did they experience a procedure? These measures are important as clinical outcomes depend upon the collaborative role a patient plays in terms of decision making and compliance. Such measures could be complemented by more objective communication measures such as a doctor’s ability to consistently transmit information about risks, benefits, and options (eg, of various treatments) to patients with a broad range of medical literacy. Other complementary objective metrics include clinical outcomes and safety. To the extent clear online metrics of an individual doctor’s outcomes or safety record exist [1], they are not currently collated by the popular doctor review websites.

The medical literature supports the idea that for some surgical procedures, surgeon volume correlates with clinical outcomes [2-11]. In other words, for specific procedures, high-volume (HV) surgeons have better results than low-volume (LV) surgeons. It is unclear why this is the case: perhaps practice makes perfect—or the more successful doctors get more referrals. But, online information about a surgeon’s volume is also hard to find—if available at all.

The question we posed was whether posts on online doctor review websites, in aggregate, correlate with surgeon volume, as a proxy for quality, for three distinct procedures. We targeted surgical procedures where this correlation has been previously suggested: lumbar surgery [12], total knee replacement [13-14], and bariatric surgery [15-18]. In other words, are high-volume surgeons, in aggregate, more likely to have positive posts (and fewer negative posts) than low-volume surgeons, in aggregate? In doing so, we hope to better understand whether high-volume doctors (who have better clinical track records overall) collectively have better online reputations.

## Methods

### Physicians

Surgeons who perform lumbar surgery, total knee replacement, and bariatric surgery were selected for study because there are data supporting a correlation between surgeon volume and clinical outcome/patient safety for each of these procedures. Further, these procedures are more likely to be considered “elective” and affect a younger demographic than vascular or oncologic procedures (for which there are also data correlating surgeon volume and clinical outcome/patient safety). We believed that “younger” patients considering an “elective” procedure would be more likely to access an online review website to help guide their decision on surgeon selection.

Current procedural terminology (CPT) codes for bariatric surgery, lumbar surgery, and total knee replacement, were identified and selected (Table 1). Although there are other codes used to label these three surgeries, the codes presented in Table 1 identify the vast majority of the patients who have had bariatric surgery, lumbar surgery, or total knee replacement.

**Table 1:** Procedure codes and selection criteria for bariatric surgery, lumbar surgery, and total knee replacement.

Procedure	Code	Description
Bariatric surgery^a^	43644	LP GSTR RSTRCIV PRC;GSTR BYPS & ROUX-EN Y
	43644	LAP GASTRIC BYPASS/ROUX-EN-Y
	43645	LP GSTR RSTRCIV PRC;GSTR BYPS&SM INTST R
	43645	LAP GASTR BYPASS INCL SMALL INTESTINE
	43770	LAP PLACE GASTR ADJ DEVICE
	43770	UNKNOWN PROCEDURE
	43770	LAPS GSTR RSTCV PX PLMT BAND
	43842	V-BAND GASTROPLASTY
	43842	GASTROPLASTY FOR OBESITY
	43843	GASTROPLASTY W/O V-BAND
	43843	GASTROPLASTY FOR OBESITY
	43846	GASTRIC BYPASS FOR OBESITY
	43846	GASTRIC BYPASS FOR OBESITY
	43846	GAST RESTRIC W/BYP; SHORT ROUX-EN-Y
	43847	GASTRIC BYPASS INCL SMALL INTESTINE
	43847	GASTRIC BYPASS FOR OBESITY
Lumbar spinal fusion^b^	22558	LUMBAR SPINE FUSION
	22612	LUMBAR SPINE FUSION
	22630	LUMBAR SPINE FUSION
	0309	OTH EXPL&DECOMPRS SPINAL CANAL
	0309	SPINAL CANAL EXPLOR NEC
	63030	LOW BACK DISK SURGERY
	63035	SPINAL DISK SURGERY ADD­ON
	63035	ADDED SPINAL DISK SURGERY
	63042	LAMINOTOMY, SINGLE LUMBAR
	63042	LOW BACK DISK SURGERY
	63044	HEMILAMINECTOMY W NERVE RT DEC
	63044	LAMINOTOMY, ADDL LUMBAR
	63047	REMOVAL OF SPINAL LAMINA
	63048	REMOVAL OF SPINAL LAMINA
	63048	REMOVE SPINAL LAMINA ADD-ON
	63056	DECOMPRESS SPINAL CORD
	63057	DECOMPRESS SPINE CORD ADD-ON
	63057	DECOMPRESS SPINAL CORD
	8108	POSTERIOR LUMBAR FUSION
	8108	LUMB LUMBOSAC FUS ANT COL POST TECH

Total knee replacement^c^	27445	REVISION OF KNEE JOINT
	27447	TOTAL KNEE ARTHROPLASTY
	27447	TOTAL KNEE REPLACEMENT
	8154	TOTAL KNEE REPLACEMENT

^a^ CPT-4 procedure codes

^b^ Mix of CPT-4 and International Classification of Diseases, 9th Revision (ICD-9) procedure codes

^c^ Mix of CPT-4 and ICD-9 procedure codes

Physician names were obtained from OptumInsight’s Normative Health Information database (NHI), a national database maintained by one of the largest aggregate insurance companies in the United States. NHI contains multi-payer, Health Insurance Portability and Accountability Act (HIPAA)-compliant transaction-level claims for more than 25 million insured patients. The Lewin Group searched the database and created a list of physicians who submitted bills at least once in 2009-2010 for the CPT codes listed for the three surgical procedures (Table 2).

**Table 2:** Number of unique physicians submitting a bill at least once to a Normative Health Insurance (NHI) carrier for relevant CPT/ICD9 procedure codes in 2009-2010^a^.

Type of surgery	Number of physicians
Bariatric surgery	1992
Lumbar spinal fusion	10,195
Total knee replacement	13,628

^a^ Database from January 1, 2010 to December 31, 2010

Our sample consisted of 600 physicians with practices in bariatric surgery (n = 200), lumbar surgery (n = 200), and total knee replacement (n = 200). From the quartile of physicians who submitted the most claims for reimbursement for each CPT/ICD9-coded target procedure, 100 physicians were randomly selected to represent “high-volume” physicians and 100 “low-volume” physicians were randomly selected from the lowest quartile of physicians (who submitted the fewest CPT/ICD9 procedure codes for reimbursement for the target procedure in 2009-2010). Low-volume surgeons submitted at least one CPT/ICD9 procedure code for the relevant procedure. The median numbers of relevant surgeries for each of the three categories performed by high- and low-volume surgeons in 2009-2010 submitting bills to a NHI carrier are reported in Table 3. The underlying supposition was that patients intending to have bariatric surgery, lumbar surgery, or total knee replacement would search the Internet for information about physicians who have the experience to perform such procedures (and submit a bill for reimbursement to an insurance company).

**Table 3:** Median number of surgical procedures performed by high- and low-volume surgeons^a^.

Type of surgery	Median surgical procedures	
	High-volume surgeons	Low-volume surgeons
Bariatric surgery	16	3
Lumbar spinal fusion	40	6
Total knee replacement	13	3

^a^ As determined by bills submitted to NHI carrier in database from January 1, 2010 to December 31, 2010.

### Data Collection

The authors were blinded as to which doctors were high-volume surgeons and which were low-volume surgeons.

Physician evaluations in the form of numerical ratings and comments were collected from 9 different heavily trafficked websites: 1 review website limits its focus to doctors and lawyers (Avvo); 3 websites limit their focus to doctors (HealthGrades, RateMDs, and Vitals); and 5 websites review a broad array of businesses and services including doctors (Citysearch, InsiderPages, Yahoo! Local, Google Maps, and Yelp). Ranking of traffic in the United States by Alexa (www.alexa.com) for the websites is presented in Table 4. Alexa is a leading provider of global web metrics, such as traffic.

**Table 4:** Alexa traffic rank in the United States for selected review websites [19].

Type of website	Website name	URL	Alexa US traffic rank
Doctor-specific			
	Avvo^a^	www.avvo.com	1613
	HealthGrades	www.healthgrades.com	570
	RateMDs	www.ratemds.com	6320
	Vitals	www.vitals.com	2029
Broad review			
	Citysearch	www.citysearch.com	341
	InsiderPages	www.insiderpages.com	1430
	Yahoo! Local	www.local.yahoo.com	4
	Google Maps	www.maps.google.com	1
	Yelp	www.yelp.com	43

^a^ Reviews lawyers also

A rating is a numerical metric defined by the patient’s subjective impression. For example, on a scale of 1-5, how does the patient rate the doctor’s overall quality, timeliness, ability to communicate, etc. Each website had different measures, but most asked at least one general question similar to: “Overall, how would you rate the doctor?”

We searched each website using the name and location of each physician in our sample. We recorded the number of ratings and the “overall” rating reported for each physician. On websites that allowed ratings on multiple dimensions (eg, communication, trust, punctuality, and time spent with patient), the averages of all numerical ratings were also recorded.

A comment is a free text description of the patient’s subjective experience. For example, “Dr. X was very compassionate and listened to each and every one of my concerns.”

We recorded the number of comments posted about each physician. One of three independent judges, also blinded to the volume of a physician’s practice, reviewed each post and categorized it as containing glowing praise or scathing criticism and whether the glowing praise or scathing criticism addressed quality of care/safety or customer service. A single post could include comments about both quality of care and customer service. If so, it was included in both counts. Comments that were neither glowing nor scathing were recorded in the total number of posts, but not in the glowing/scathing tallies. A prototypical example of a glowing quality of care/safety comment is “Dr. X gave me back my life.” In comparison, a scathing quality of care/safety comment is “Dr. X was a butcher.” A prototypical example of a glowing customer service comment is “Dr. X returned my call late at night and gave me all the time I needed.” In comparison, a scathing customer service comment is “Dr. X was dismissive, arrogant, and never listened.” One of the websites, HealthGrades, does not allow posting of comments.

Since many consumers may not do an exhaustive search for physician information, we recorded whether a link to any of the study websites was among the first 20 retrieved in a Google search for each physician in the lumbar and total knee replacement samples. A Google search was performed on each doctor in each of three formats:

1. “Dr. First_Name Last_Name” + “City, State”

2. “First_Name Last_Name, D.O.” + “City, State”

3. “Dr. First_Name Last_Name, M.D.” + “City, State”

Separate analyses were performed using only data retrieved in this abbreviated search. The first 20 links correlate with the first 2 webpages retrieved in a typical search as the default setting for a Google search is 10 results per page. [20]

Once the data was captured from the online review websites, the spreadsheet was sent to the Lewin Group. They added a field indicating whether a doctor was high volume or low volume. All other physician-identifying information was subsequently stripped and the rows were shuffled. The database was then returned to the authors for analysis.

### Analytic Approach

Do ratings and comments posted on physician review websites provide valid information regarding surgical volume, a proxy for clinical outcomes/safety? We answered this by comparing the information available on high- and low-volume physicians, controlling for surgical practice in a 2 × 3 analysis of variance. Our analysis also considered whether the differences between high- and low-volume physicians were consistent across bariatric, lumbar, and total knee replacement surgical practices.

Analyses were performed using the mean number of ratings per website (on which each physician was rated at least once). Additional analyses were performed for each physician’s overall rating, averaged across websites. Analyses using physicians’ overall ratings tracked averages that included ratings of specific physician characteristics (average of multidimensional numerical ratings) very closely (all *r* > .85), so only analyses using the overall rating are presented. The Vitals website uses a different rating scale (1-4) than the other websites (1-5); therefore, ratings from each website were standardized using a *z* test (converting each physician’s score into a value expressed as the number of standard deviations from the mean on each website). The *z* score, or standard score, allowed for averaging ratings across websites.

Analyses were performed using the average number of comments per physician on websites with at least one posted comment. Additional analyses were performed identifying the proportions of comments that were glowing and scathing broken down by whether they concerned the physicians’ quality of care or customer service.

## Results

First, we report the results of these analyses using all available data for each physician. Second, we report analyses restricted to data available in the first 20 links of a Google search for each physician in the lumbar surgery and total knee replacement samples. Finally, we present the results of an analysis that explores the incremental validity of using data from both ratings and posted comments to distinguish high- and low-volume physicians.

Table 5 presents the numbers of physicians in our sample with ratings and comments posted on each of the study websites.

**Table 5:** Numbers of surgeons with ratings and comments posted on a study website.

	Surgeons with ratings (N = 547)				Surgeons with comments (N = 385)			
Website	Bariatric (n = 170)	Lumbar (n = 182)	Knee (n = 195)	Total	Bariatric (n = 101)	Lumbar (n = 147)	Knee (n = 137)	Total
Vitals	101	134	137	372	68	107	98	273
HealthGrades	129	161	165	455				
RateMDs	44	91	77	212	40	87	74	201
InsiderPages	100	141	151	392	7	9	13	29
Avvo	1	2	3	6	1	2	3	6
Yahoo! Local	11	17	20	48	10	17	19	46
Google Maps	9	16	6	31	1	14	5	20
Citysearch	2	5	3	10	2	2	1	5
Yelp	3	1	4	8	3	1	4	8
Total	400	568	566	1534	132	239	217	588

### All Available Data

Numerical ratings were found for the majority (547/600, 91.2%) of the physicians in our sample; comments were found for 385 (64.2%) of the physicians. The average physician had ratings on 3 of the 9 websites (range: 1-7) and comments on 1 website (range: 1-5). Preliminary analysis noted the correlation between rank orders of physicians’ total number of ratings aggregated across all websites and total number of ratings per website was *r* = .86, (*P* < .001). Additional preliminary analyses revealed that high-volume physicians had more total ratings across all websites and ratings on more websites than did low-volume physicians. Our analyses focus on average number of ratings per website on which a physician is rated—based on an assumption that a typical consumer may not do an exhaustive review of all available ratings on many websites but be satisfied upon finding one website with information on his or her physician.

Table 6 presents results of analyses of all available physician data. High-volume physicians had significantly more ratings per website compared to low-volume physicians for every type of practice (*P* < .001) and there was no evidence that this effect differed among physician groups (*P* = .15). However, the standardized numerical ratings assigned to high-volume physicians were not significantly different from those assigned to low-volume physicians (*P* = .27), nor was this null finding different across physician groups (*P* = .48). Table 6 also shows that high-volume physicians had more comments per website than did low-volume physicians for each type of practice (*P* = .05). Again, there was no evidence this differed among physician groups (*P* = .74).

Table 7 shows that only comments related to quality of care seem to distinguish high- and low-volume physicians; high-volume physicians had a significantly greater proportion of glowing comments (*P* = .002) and a significantly lower proportion of scathing comments regarding quality of care than low-volume physicians (*P* = .005). Again, we observe these patterns for each surgical practice and our analyses offer no basis for inferring that it’s more true for one group than another (*P* = .70 for glowing; *P* = .41 for scathing). We also observed that there were far more glowing than scathing comments overall, even for low-volume physicians. In general, we observed that high-volume physicians tend to have almost 64% glowing comments (versus 51% for low-volume physicians) regarding quality of care. Proportion of glowing/scathing comments related to customer service did not differentiate between high- versus low-volume physicians overall (*P* = .52 for glowing; *P* = .48 for scathing) nor was there evidence that this null finding differed across physician groups (*P* = .92 for glowing; *P* = .20 for scathing).

**Table 6:** Analysis of ratings and comments for high- and low-volume surgeons.

		Surgeon volumes									Analysis of variance					
		All		Bariatric		Lumbar		Knee		Volume^a^		Procedure^b^		Interaction^c^	
		HV	LV	HV	LV	HV	LV	HV	LV	*F*	*P*	*F*	*P*	*F*	*P*
**Surgeons with ratings**																
	N=	547		170		182		195		*F1,541*		*F2,541*		*F2,541*	
	Mean ratings/ website^d^ (SD)	5.85 (3.92)	4.57 (3.29)	4.40 (3.22)	3.70 (2.53)	7.49 (4.29)	5.39 (3.97)	5.60 (3.58)	4.63 (3.02)	18.33	<.001	20.73	<.001	1.88	.15
	Overall rating score^e^	0.07 (0.74)	-0.00 (0.84)	0.35 (0.68)	0.19 (0.90)	-0.14 (0.75)	-0.10 (0.85)	0.03 (0.69)	-0.07 (0.77)	1.21	.27	11.98	<.001	0.74	.48

**Surgeons with comments**																
	N=	385		101		147		137		*F1,379*		*F2,379*		*F2,379*	
	Mean comments/ website^d^ (SD)	2.74 (1.95)	2.30 (2.05)	2.03 (1.30)	1.78 (1.36)	3.07 (2.00)	2.74 (2.44)	2.87 (2.15)	2.25 (2.01)	3.82	.05	7.72	.001	0.30	.74

^a^ Comparing high- versus low-volume surgeons

^b^ Comparing bariatric, lumbar, and knee surgeons

^c^ Comparing high- versus low-volume surgeons across surgeon categories

^d^ Only includes individual websites on which doctor had at least one rating/comment

^e^ *z* score

**Table 7:** Analysis of scathing and glowing comments for high- and low-volume surgeons.

		Surgeon types										Analysis of variance					
		All (N = 385)			Bariatric (n = 101)			Lumbar (n =147)		Knee (n = 137)		Volume^a^		Procedure^b^		Interaction^c^	
		HV		LV	HV	LV		HV	LV	HV	LV	*F 1,379*	*P*	*F 2,379*	*P*	*F 2,379*	*P*
**Quality of care comments**																	
	Glowing mean (SD)		1.76 (1.56)	1.25 (1.46)	1.09 (0.98)	0.73 (0.64)	2.06 (1.50)		1.55 (1.50)	1.89 (1.81)	1.35 (1.76)	9.43	.002	11.59	<.001	0.11	.89
	Glowing % (SD)		0.64 (0.35)	0.51 (0.38)	0.53 (0.38)	0.44 (0.41)	0.70 (0.30)		0.55 (0.34)	0.64 (0.36)	0.53 (0.40)	9.87	.002	4.57	.01	0.36	.70
	Scathing mean (SD)		0.35 (0.65)	0.44 (0.57)	0.26 (0.49)	0.34 (0.57)	0.40 (0.79)		0.57 (0.61)	0.35 (0.59)	0.38 (0.53)	1.98	.16	2.80	.06	0.42	.66
	Scathing % (SD)		0.14 (0.26)	0.23 (0.34)	0.15 (0.30)	0.19 (0.35)	0.12 (0.22)		0.27 (0.33)	0.15 (0.27)	0.23 (0.35)	8.01	.005	0.28	.76	0.90	.41
														
**Customer service comments**																	
	Glowing mean (SD)		0.65 (0.90)	0.52 (0.89)	0.23 (0.41)	0.17 (0.42)		0.60 (0.72)	0.57 (1.02)	1.00 (1.16)	0.74 (0.93)	1.65	.20	17.83	<.001	0.74	.48
	Glowing % (SD)		0.24 (0.30)	0.22 (0.32)	0.11 (0.23)	0.09 (0.26)		0.22 (0.27)	0.22 (0.31)	0.36 (0.33)	0.32 (0.35)	0.41	.52	18.93	<.001	0.09	.92
	Scathing mean (SD)		0.58 (0.87)	0.49 (0.79)	0.32 (0.60)	0.18 (0.42)		0.73 (0.93)	0.67 (0.93)	0.60 (0.92)	0.56 (0.80)	0.97	.32	9.29	<.001	0.12	.89
	Scathing % (SD)		0.19 (0.27)	0.21 (0.32)	0.14 (0.28)	0.08 (0.21)		0.22 (0.27)	0.26 (0.32)	0.19 (0.26)	0.26 (0.36)	0.49	.48	6.78	.001	1.60	.20

^a^ Comparing high- versus low-volume surgeons

^b^ Comparing bariatric, lumbar, and knee surgeons

^c^ Comparing high- versus low-volume surgeons across surgeon categories

### First 20 Links

We conducted a reanalysis of the physician data restricted to review websites within the first 20 links returned by a Google search of a physician’s name (Table 8). These searches returned links to some or all of our sample doctor review websites enabling access to the majority (896/1134, 79%) of webpages where doctors had at least one rating and of the webpages where doctors had at least one comment (347/456, 76%). This analysis was restricted to lumbar and total knee replacement samples. We excluded bariatric surgery from this subanalysis because the number of reviews and comments accessible via the first 20 links for that category was inadequate to draw meaningful conclusions. The analyses in Table 9 parallel those reported in Table 7 using the full available data.

Again, we find that high-volume physicians had greater numbers of ratings and comments per linked website than did low-volume physicians. The numerical ratings given to high- and low-volume physicians did not differ. And high-volume physicians had greater proportions of glowing (and lower proportions of scathing) comments about quality of care than did low-volume physicians. There were no differences in proportions of comments concerning customer service.

**Table 8:** Analysis of ratings and comments for high- and low-volume surgeons on first 20 websites (excluding bariatric surgery).

		Surgeon volumes						Analysis of variance					
		All (N = 374)		Lumbar (n = 181)		Knee (n = 193)		Volume^a^		Procedure^b^		Interaction^c^	
		HV	LV	HV	LV	HV	LV	*F1,370*	*P*	*F1,370*	*P*	*F1,370*	*P*
**Ratings**													
	Mean ratings/ website^d^ (SD)	6.76 (4.18)	5.47 (4.55)	7.70 (4.39)	6.08 (5.24)	5.85 (3.76)	4.93 (3.78)	8.21	.004	11.30	.001	0.62	.43
	Overall rating score^e^ (SD)	-0.01 (0.78)	0.02 (0.83)	-0.08 (0.80)	0.01 (0.85)	0.05 (0.76)	0.04 (0.81)	0.22	.64	0.96	.33	0.38	.54
**Comments**													
		Surgeon volumes						Analysis of variance					
		All (N = 266)^a^		Lumbar (n = 138)		Knee (n = 128)		Volume^a^		Procedure^b^		Interaction^c^	
		HV	LV	HV	LV	HV	LV	*F1,262*	*P*	*F1,262*	*P*	*F1,262*	*P*
	Mean comments/ website^d^ (SD)	3.16 (2.36)	2.51 (2.32)	3.24 (2.24)	2.71 (2.60)	3.06 (2.51)	2.32 (2.03)	4.78	.03	0.98	.32	0.13	.72

^a^ Comparing high- versus low-volume surgeons

^b^ Comparing lumbar and knee surgeons

^c^ Comparing high- versus low-volume surgeons across surgeon categories

^d^ Only includes individual websites on which doctor had at least one rating/comment

^e^ *z* score

**Table 9:** Analysis of scathing and glowing comments for high- and low-volume surgeons on first 20 websites (excluding bariatric surgeons).

		Surgeon volumes						Analysis of variance					
		All (N = 266)		Lumbar (n = 138)		Knee (n = 126)		Volume^a^		Procedure^b^		Interaction^c^	
		HV	LV	HV	LV	HV	LV	*F1,262*	*P*	*F1,262*	*P*	*F1,262*	*P*
**Quality of care comments**													
	Glowing mean (SD)	2.06 (1.74)	1.44 (1.72)	2.13 (1.59)	1.52 (1.67)	1.98 (1.92)	1.36 (1.77)	8.11	.005	0.51	.48	0.00	.99
	Glowing % (SD)	0.68 (0.33)	0.53 (0.40)	0.71 (0.30)	0.55 (0.38)	0.65 (0.36)	0.52 (0.42)	10.76	.001	0.78	.38	0.12	.73
	Scathing mean (SD)	0.43 (0.88)	0.44 (0.60)	0.45 (0.87)	0.52 (0.62)	0.39 (0.89)	0.37 (0.57)	0.04	.84	1.24	.27	0.22	.64
	Scathing % (SD)	0.12 (0.23)	0.22 (0.34)	0.12 (0.22)	0.25 (0.35)	0.12 (0.24)	0.19 (0.33)	7.34	.007	0.76	.39	0.74	.39
													
**Customer service comments**													
	Glowing mean (SD)	0.80 (0.99)	0.73 (1.05)	0.60 (0.81)	0.59 (1.09)	1.03 (1.13)	0.87 (1.00)	0.48	.49	8.13	.005	0.397	.53
	Glowing % (SD)	0.29 (0.32)	0.31 (0.36)	0.22 (0.29)	0.23 (0.32)	0.38 (0.34)	0.38 (0.39)	0.01	.94	13.93	<.001	0.04	.85
	Scathing mean (SD)	0.71 (1.07)	0.61 (0.90)	0.81 (1.04)	0.69 (0.98)	0.60 (1.10)	0.53 (0.82)	0.61	.44	2.24	.14	0.03	.86
	Scathing % (SD)	0.19 (0.26)	0.24 (0.33)	0.22 (0.27)	0.26 (0.32)	0.16 (0.24)	0.23 (0.35)	2.13	.15	1.38	.24	0.16	.69

^a^ Comparing high- versus low-volume surgeons

^b^ Comparing lumbar and knee surgeons

^c^ Comparing high- versus low-volume surgeons across surgeon categories

### Additional Analyses

The preceding analyses suggest that high- and low-volume surgeons could be identified based on the (1) number of ratings; (2) number of comments; (3) proportion of glowing comments about quality of care; and (4) proportion of scathing comments about quality of care. Next, we attempted to establish the practical usefulness of these various pieces of information for distinguishing high- and low-volume physicians. The (discriminant) analysis develops a function that maximally distinguishes study groups from each other. Function coefficients (see Table 10) are the weights that support this discrimination; higher absolute weights indicate greater contribution of the variable to differentiating groups from each other. As illustrated in the table, discriminant analysis suggests that ratings per website, and proportion of glowing comments about quality of care are the two most differentiating pieces of information (highest absolute weights), followed by proportion of scathing comments about quality of care. The number of comments per website, while providing some information when examined alone, provides little additive information (beyond the other measures).

As a follow-up, we also performed a classification analysis wherein physicians’ surgical volume (high or low) was “predicted” by the number of ratings and comments they received as well as the proportion of glowing and scathing comments about quality of care (using the discriminant function). The results revealed that one could accurately identify a physicians’ surgical volume 61.6% of the time. An examination of the resulting discriminant function revealed that the number of ratings per website and proportion of glowing postings seemed most central to the discrimination, followed by proportion of scathing comments. Number of comments was largely redundant to these other measures.

**Table 10:** Discriminant function analysis results.

	Standardized function coefficients
Ratings per website	0.57
Comments per website	0.08
Proportion glowing (quality of care)	0.46
Proportion scathing (quality of care)	-0.35
	
Discriminant function is significant ( = 21.4, *P* < .001)	

## Discussion

Our study found there is evidence that online doctor review websites can be used to identify high-volume surgeons performing targeted procedures—a proxy which correlates with higher quality care. Patients naturally want to identify, and be treated by, the best practitioners. And they seek such information online. The importance of the Internet in determining patients’ health care choices in the United States should not be underestimated. A recent study by The Pew Internet and American Life Project noted that 59% of adults have looked online for information on 15 health topics such as a specific disease or treatment [21]. And they are looking for information about health care providers too; 12% of adults have consulted online rankings or reviews of doctors or other providers.

Online review websites track patient sentiment. Recent advances even allow for automating the classification of patient comments by sentiment. Xia et al [22] described a multistep sentiment classifier for patient opinion mining that, in principle, could analyze large collections of data, online or otherwise, to assign sentiment scores to patient reviews. While patient sentiment is helpful, to our knowledge, our study is the first to tackle the connection between patient reviews, patient sentiment, and a proxy for clinical outcomes.

Defining quality in healthcare is difficult. From a patient’s perspective, soft measures (eg, communication skills and ability to listen) are important for issues such as decision making and compliance—issues which impact outcomes. More objectively, quality often distils to patient safety and clinical outcomes. Such metrics include morbidity and mortality rates, length of stay in hospital, blood loss, time to return to work, and the like. This detailed information tracking of individual practitioners is not readily available online for patients to analyze.

The medical literature suggests that, for a number of surgical procedures, the volume of cases performed annually by an individual surgeon correlates with patient safety and clinical outcome metrics. In other words, for specific procedures, high-volume surgeons have better results than low-volume surgeons do.

We targeted three surgical procedures where this correlation has been shown previously: lumbar surgery [12], total knee replacement [13-14], and bariatric surgery [15-18]. To our knowledge, our analysis is the first to tackle the question of whether online reviews can identify the more successful surgeons using a proxy for clinical outcomes and safety. We posed the following hypothetical question: Do quantity and character of posts on online doctor review websites, in aggregate, correlate with surgeon volume, as a proxy for quality, for these three distinct procedures?

Our findings provide evidence that the following data aggregated from 9 doctor review websites can distinguish high-volume from low-volume surgeons: total number of numerical reviews; total number of text comments; proportion of glowing positive comments; and proportion of scathing negative comments. Analysis of the actual numerical ratings did not distinguish between high- and low-volume surgeons. The same conclusions were noted when limited to doctor review websites from the first 20 links of a Google search for the doctor’s name.

While our analysis provides evidence that data from doctor review websites can help consumers identify higher quality doctors, the effect size is weak. From the patient’s perspective, a far better way to determine whether a surgeon performs a high volume of procedures is to ask the doctor. Or the doctor could preemptively provide such information on the various review websites.

One surprising result was while the total number of reviews correlated with surgeon volume, the actual rating value did not. Also, it is unclear why the total number of reviews and comments are associated with surgeon volume. Perhaps high-volume surgeons are more comfortable with their skills/results and are more likely to ask their patients for feedback—internally or on the Internet. In any event, such observations deserve further study.

Our analyses also supported a finding previously reported by others [23]; namely, on online review websites, the single metric (overall rating) correlated highly with more granular, multidimensional numerical ratings. In our analyses, this correlation was between overall rating and the average of all multidimensional ratings (all *r* > .85). Accordingly, analyzing patient responses to the question “Overall, how would you rate this doctor?” predicts positive and negative sentiment from more detailed questions.

Even with these findings, it is still an open question whether consumers should rely heavily on the websites partly because the websites have limited data. Among the 600 doctors, on websites where the doctor was rated, the average doctor had between 4 and 6 ratings and between 2 and 3 comments. As the websites accumulate more data, our conclusions may change.

Our study identified at least one rating for 91% of doctors in our sample. This contrasts with the study by Lagu et al [24] where 70% of their physician sample did not have a single review on any of the 33 websites they looked at. This study captured data limited to Boston generalists and undefined subspecialists in the spring of 2009. Our study captured data for specific categories of surgeons across the country in the summer of 2011. The experience a patient has with a surgeon is arguably different from the experience one has with a generalist or many types of subspecialists. The surgical experience is typically a “once-off.” The experience with a generalist and many types of subspecialists is typically longer term. Patients may be more inclined to post ratings and comments based on a single (more emotionally charged) experience with a surgeon compared with a routine long-term experience with a generalist. But, the threshold of a doctor converting from no reviews on any website to at least one review on a website is low. The average doctor sees over 1000 patients per year. If just one patient takes the effort to post a review, that threshold is crossed. As our data was gathered two years after that of Lagu et al, this suggests that although the number of online reviews per doctor is still limited, the trend is for more reviews for more doctors.

Our study was limited to a sample of targeted surgical procedures. Within that dataset, there may be high-volume surgeons who have poor clinical outcomes/patient safety records. And there may be low-volume surgeons with excellent clinical outcomes/patient safety records. Our study only attempted to track a proxy for clinical quality—surgical volume—and not clinical quality itself. Also, our sample makes no conclusions about surgeons who perform procedures other than those analyzed or any conclusions about non-surgical practitioners.

Another limitation is that the NHI database used to identify low- and high-volume surgeons, while extensive, only covered CPT/ICD9 procedure codes submitted to private insurance carriers. The NHI database does not reflect data submitted to Medicare. In surveying the literature correlating surgeon volume with quality of care, we intentionally selected three surgical procedures that were more likely than others to be performed on a younger demographic, hoping to minimize whatever effect the absence of Medicare data might have on our analysis.

One further limitation is that our classification of comments into the categories of quality of care and customer service as glowing praise or scathing criticism required human judgment, making it susceptible to potential inter-reviewer variance. While it is unlikely different reviewers would classify words such as “butcher” and “life saver” differently, new technologies [22] may help automate the review process for greater consistency.

Online doctor review websites provide a growing collection of data for consumers to use. These websites provide fertile ground for future studies on whether its data can help patients reliably differentiate doctors who provide better clinical outcomes and patient safety.

In summary, online review websites provide a rich source of data that may be able to track quality of care, though the effect size is weak and not consistent for all review website metrics.

## Abbreviations

CPT: current procedural terminology
HV: high volume
ICD9: International Classification of Diseases, 9th Revision
LV: low volume
NHI: Normative Health Information
HIPAA: Health Insurance Portability and Accountability Act
